# Could Statin Therapy Be Useful in Patients With Coronavirus Disease 2019 (COVID-19)?

**DOI:** 10.3389/fcvm.2021.775749

**Published:** 2021-10-27

**Authors:** Jose D. Torres-Peña, Niki Katsiki, Pablo Perez-Martinez

**Affiliations:** ^1^Lipids and Atherosclerosis Unit, Department of Internal Medicine, Maimonides Biomedical Research Institute of Cordoba (IMIBIC), Reina Sofia University Hospital, University of Cordoba, Cordoba, Spain; ^2^CIBER Fisiopatología de la Obesidad y Nutrición (CIBEROBN), Instituto de Salud Carlos III, Cordoba, Spain; ^3^Division of Endocrinology and Metabolism, First Department of Internal Medicine, Diabetes Center, AHEPA University Hospital, Aristotle University of Thessaloniki, Thessaloniki, Greece

**Keywords:** coronavirus disease (COVID-19), statins, acute respiratory disease syndrome, inflammation, death

## Abstract

Acute respiratory distress syndrome (ARDS), resulting from an exaggerated inflammatory response, is the main cause of death from the coronavirus disease 2019 (COVID-19). Apart from respiratory infection, COVID-19 patients can develop cardiovascular disorders such as myocardial injury and myocarditis, pericarditis, cardiac arrest and arrhythmias, cardiomyopathy, heart failure, coagulation abnormalities and thrombosis. Statins can beneficially affect inflammation, oxidative stress, coagulation, thrombosis, angiotensin converting enzyme receptor, lipid rafts, and endothelial function. In this narrative review, we provide a critical overview of the current evidence and future perspectives on the use of statins to modulate the severity, duration and complications of COVID-19 through their pleiotropic properties.

## Introduction

The coronavirus disease 2019 (COVID-19) involves the development of severe acute respiratory distress syndrome (ARDS) caused by infection from the severe acute respiratory syndrome coronavirus 2 (SARS-CoV-2) (1). COVID-19 has been characterized as a pandemic health emergency from the World Health Organization since 30 January 2020 (2). The main clinical manifestation of COVID-19 is pulmonary infection, with ARDS being the major cause of death, resulting from an exaggerated inflammatory response (3).

Apart from the respiratory system, SARS-CoV-2 infection can also harm the cardiovascular (CV) system, leading to the development of cardiac abnormalities such as myocardial injury, myocarditis-pericarditis, arrhythmias, cardiac arrest, cardiomyopathy, heart failure and cardiogenic shock (4). An increased risk for coagulation disorders, including venous thromboembolism (VTE) and disseminated intravascular coagulation (DIC), has also been reported in relation to COVID-19 (5). Several pathophysiological mechanisms are implicated in this process, including angiotensin-converting enzyme 2 (ACE2)-dependent pathways (with ACE being the receptor for SARS-CoV-2 that facilitates viral entry and replication), cytokine storm activation, hypoxemia, drug toxicities, stress-induced vascular dysfunction, thrombocytopenia, prolonged prothrombin time and fibrinolysis (4–6). Statins are the first-line therapy for treating dyslipidemias and CV risk. Apart from their lipid-lowering properties, statins also exert pleiotropic benefits on inflammation, oxidative stress, coagulation and endothelial function (7). Such beneficial effects may modulate the severity, duration, and complications of COVID-19. In this narrative review, we provide an overview of the current evidence and future perspectives on the use of statins in COVID-19 patients.

## Statins Mechanisms of Action Involved in SARS-CoV-2 Infection

### Angiotensin Converting Enzyme 2 (ACE2) Receptor and SARS-CoV-2 Infection

It is important to understand the interaction of the SARS-COV-2 with the host cell. This coronavirus is a RNA virus with a lipid envelope. The virus genome encodes four structural proteins: the membrane protein, the nucleocapsid protein, the envelope protein, and the spike protein, which mediates attachment to the ACE2 (8). SARS-CoV-2 binds to the ACE2 protein on the surface of the lung cells and enters intracellularly; once infected, the cells reduce (9). Since ACE2 breaks down angiotensin II, a pro-inflammatory factor, it has been postulated that ACE2 downregulation is responsible for at least some of the inflammatory effects induced by SARS-COV-2 infection through the predomination of angiotensin II both locally (in the lungs) and systemically (10).

Statins can increase ACE2 levels (11). In this context, atorvastatin increased ACE2 level in the kidneys and heart in animal models with atherosclerosis (12). These effects have also been reported with pravastatin (13) and rosuvastatin (14). These findings support the hypothesis that statins inhibit RAS activation, improve vascular remodeling after vascular injury and reduce angiotensin II pro-inflammatory effects. Therefore, statin use can be beneficial in the course of an inflammatory disease such as COVID-19 (15).

### CD147 Cell Surface Protein and SARS-CoV-2 Infection

CD147 is a surface protein that can act as a coronavirus receptor (16). CD147 is overexpressed in cancer, atherosclerosis and infectious diseases, including the SARS-CoV-1 and SARS-CoV-2. Wang et al. (17) first discovered an interaction between cell receptor CD147 and SARS-CoV-2 spike protein, modulating virus cellular entry by endocytosis. In their study, they showed that loss or blocking of CD147 in *in vitro* cell lines by an anti-CD147 antibody, namely mepolizumab, inhibited SARS-CoV-2 amplification.

Statins can alter CD147 expression, structure and function by inhibiting its isoprenylation and N-glycosylation (18). In particular, in cultured monocytes that received a pretreatment with atorvastatin, pravastatin, or fluvastatin, CD147 translocation to cell surface was impaired, resulting in a inhibition of differentiation to macrophages (18). This finding highlighted the ability of statins for immunomodulation. Furthermore, this statin-induced suppression of CD147 expression can inhibit the entry of SARS-CoV-2 within the host cells, thus supporting an antiviral role for statins by decreased SARS-CoV-2 infectivity.

### Toll-Like Receptors-Myeloid Differentiation Primary Response 88 Pathways and SARS-CoV-2 Infection

Toll-like receptors (TLRs) are proteins that in animal models can reduce ARDS (19). In contrast, the gene expression of myeloid differentiation primary response 88 (MyD88) downregulate TLRs and it's known to be increased in the SARS-CoV infections (20). Dysregulation of MyD88 results in NF-kB activation that can increase lung damage (21), thus, statins preserve MyD88 at normal levels during infection and reduce NF-kB activation and it has been proposed that the modulation of TRL-MyD88 pathway by statins may be useful to treat COVID-19. Other authors suggest that secondary compensatory immune response due to TRL-NF-kB downregulation may exert poor COVID-19 outcomes (22). In summary, the role of interaction between statins and TLR-MyD88 pathways is controversial.

### Lipids Raft and SARS-CoV-2 Infection

Cell membranes have a lipid bilayer that contains subdomains rich in sphingolipids and cholesterol, named lipids rafts. Lipid rafts host several receptors involved in inflammatory and immune responses and play a key role as a “point of entry” for many viruses such as Influenza, Ebola or human immunodeficiency virus (HIV)-1 (23–25). In coronavirus infection, they specifically promote the interaction between the S protein and ACE2 receptor, thus facilitating viral endocytosis (26). Overall, cellular cholesterol has been suggested as a core contributor to SARS-CoV-2 viral entry and therefore, cholesterol depletion and lipid rafts disruption may decrease viral infection, by inhibiting the entry of the virus into the host cells (27). Furthermore, viral replication involves the lipid metabolism which is upregulated by viruses to meet the increased demand for viral structural elements (e.g., for the viral cell membrane) (28). Thus, drugs that suppress cholesterol synthesis, e.g., statins, could be helpful, both by avoiding early infectivity and inhibiting viral replication (29).

### Inflammasome in SARS-CoV-2 Infection

The inflammasome is a multiprotein complex that mediates the activation of caspase-1, leading to the activation of interleukin (IL)-1β in a variety of diseases, such as the Alzheimer's disease, Parkinson's disease, CV and renal diseases, rheumatoid arthritis and infections by bacteria or viruses (30). Different inflammasomes have been described: NLRP1, NLRP2, NLRP3, NLRC4, and AIM2, but the most studied up to the date is the NLRP3. There is evidence implicating the NLRP3 inflammasome and IL-1β in mediating inflammation during lung injury and ARDS (31). During the SARS-CoV-2 infection, NLRP3 stimulation has been described, inducing cell death (32). Inflammasome activation in SARS-CoV-2 results in respiratory, CV, gastrointestinal, neurological, renal and ophthalmic manifestations (30).

Statins have been shown to suppress NLRP3 inflammasome activation (33), although conflicting results have also been reported (34, 35). Further research is needed to elucidate the effects of statins on the NLRP3 inflammasome.

### Coagulation Abnormalities and Thrombosis in SARS-CoV-2 Infection

The most frequent findings in patients with COVID-19 and coagulopathy are increased D-dimer, decreased platelet count and a prolongation of the prothrombin time (36). In one of the first series of patients infected with SARS-CoV-2, elevated D-dimer levels were observed in the 46% of the patients (37). Furthermore, thrombocytopenia can be found in 70–95% of patients with severe COVID-19 (38). Venous thromboembolism may also occur in patients with severe COVID-19 infection (39). Indeed, in post-mortem evaluation in COVID-19 patients, thrombotic depositions were observed in small vessels of the lungs and other organs (40). Such coagulation abnormalities related to severe COVID-19 infection can lead to respiratory deterioration and death (39). Statins have been reported to significantly reduce the rate of 30-day non-fatal myocardial infarction and death in patients with acute coronary syndrome in the Platelet Receptor Inhibition in Ischemic Syndrome Management (PRISM) study (*n* = 1,616) (41). Furthermore, in the Stroke Prevention by Aggressive Reduction in Cholesterol Levels (SPARCL) trial (*n* = 4,731) (42) atorvastatin significantly decreased the incidence of fatal or non-fatal stroke, as well as major CV events in patients with recent stroke or transient ischemic attack (TIA), irrespective of baseline ischemic stroke subtype. Therefore, statins can prevent atherothrombotic events. There is also experimental data supporting a direct antithrombotic effect of statins via inhibition of several pathways of hemostasis, including coagulation cascade and platelet activation (43). Anticoagulant properties of statins involve reduced thrombin generation by decreased tissue factor expression, and thus, attenuation of pro-coagulant reactions catalyzed by thrombin, such as factor V and factor XIII activation and fibrinogen cleavage, as well as increased protein C activation and factor Va inactivation via enhanced endothelial thrombomodulin expression (44). Furthermore, statins can deactivate platelets via cholesterol-lowering dependent and independent mechanisms (45). Finally, statins may protect against venous thromboembolic events (VTE) [e.g., pulmonary embolism (PE) and deep vein thrombosis (DVT)] via both anticoagulant and anti-inflammatory actions (46). Indeed, several clinical studies support statin-induced protection from VTE (47). Of note, previous meta-analyses showed that statins can decrease plasma D-dimer levels (48). Such statin effects may proven beneficial in modulating coagulation in patients with SARS-CoV-2 infection. In this context, the ongoing Intermediate vs. Standard-dose Prophylactic anticoagulation In cRitically-ill pATIents with COVID-19: An open label randomized controlled trial (INSPIRATION) and INSPIRATION-statin (INSPIRATION-S) studies will elucidate the effects of antithrombotic and thromboinflammatory therapy in critically-ill patients with COVID-19 (49).

### Endothelial Dysfunction in SARS-CoV-2 Infection

SARS-CoV-2 affects vascular endothelium in different organs such as the heart, lung, kidneys, gastrointestinal tract or liver (50). In this context, the endothelial injury observed in patients infected by SARS-CoV-2 that can result into systemic endothelitis may be attributed to a combination of direct virus infection and an exaggerated immune and inflammatory response, leading to artery contraction and thrombus formation (24). Indeed, endothelial dysfunction may aggravate during the course of the COVID-19, impair organ perfusion and promote coagulation, thus predisposing patients to both micro- and macro-vascular thrombotic events (51). The Working Group on Atherosclerosis and Vascular Biology together with the Council of Basic Cardiovascular Science of the European Society of Cardiology recently published a position statement on the role of the endothelium in the pathophysiology of COVID-19 (52).

Statins improve endothelial dysfunction in patients with or at risk for CV disease (53). Several mechanisms are involved in this process, including endothelial nitric oxide synthase overexpression, inhibition of pro-inflammatory pathways, reduction of oxidized low-density lipoprotein, platelet deactivation and antithrombotic properties (54, 55). These statin-induced effects can be beneficial in COVID-19 patients but further research in this field in needed.

**Figure 1:**
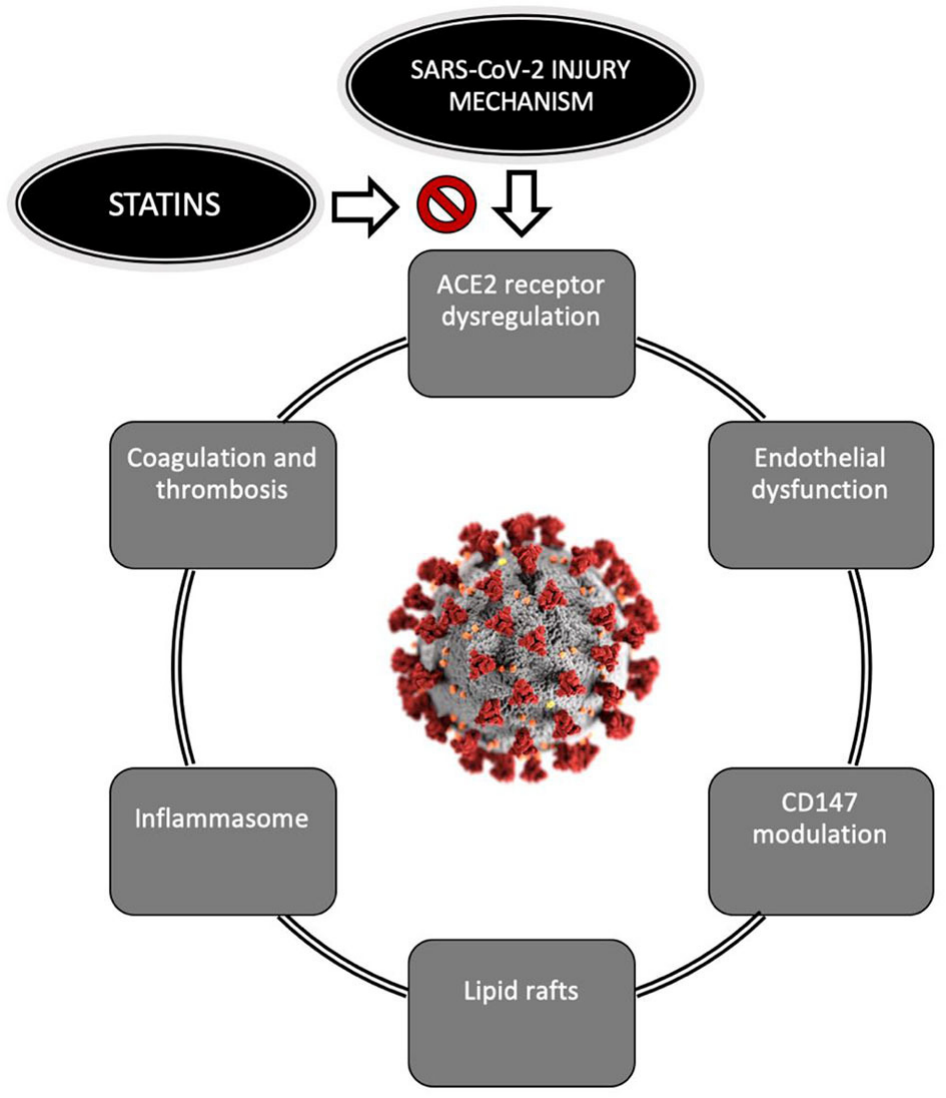
Pathophysiological mechanisms of COVID-19 which statins may affect. COVID-19, Coronavirus disease; CDC, Center for Disease Control. The SARS-CoV-2 image was downloaded from the CDC public library (https://phil.cdc.gov).

In summary, there is evidence for a potential role of statins as modulatory drugs with beneficial effects on different infectious processes of viral origin via their ability to interact with the ACE2 receptor, inhibit CD147 surface receptor, affect lipid rafts, suppress the NLRP3 inflammasome, inhibit coagulation and improve endothelial function. Based on these actions, statins may protect against the incidence and progression of SARS-CoV-2 infection (Figure 1).

## Effects of Statins on SARS-CoV-2 Infection Outcomes

The adverse effects of COVID-19 were initially thought to be limited to the respiratory tract, but it has been shown that the virus affects most of the organs and thus, COVID-19 may be considered a systemic entity (52). Adverse outcomes include ARDS, acute renal failure, myocardial damage and death (52). In this section, the impact of statins on COVID-19 outcomes is discussed.

SARS-CoV-2 infection can progress to a severe form of ARDS, requiring mechanical ventilation. As previously mentioned, the pathogenesis of ARDS in closely related to endothelial dysfunction, hypercoagulability and dysregulated inflammatory response (3). Zhang et al. (56) performed a retrospective study on 13,981 patients with COVID-19 in Hubei (China), among which 1,219 received statins. After matching baseline differences in two groups comparing those who received statins with those not on statins, a lower incidence of invasive mechanical ventilation (IMV) [adjusted hazard ratio (aHR) 0.51, 95% confidence interval (CI): 0.34–0.78; *p* = 0.002], ARDS (aHR 0.59, 95% CI: 0.37–0.92; *p* = 0.020) and acute cardiac injury (aHR 0.61, CI: 0.38–0.97; *p* = 0.038) was observed in statin users vs. non-users (30). In another retrospective cohort with 249 patients hospitalized with COVID-19, statin use was associated with a reduced risk for IMV [odds ratio (OR) 0.45, 95% CI: 0.20–0.99) (57). Similarly, we recently showed that prior statin treatment that is maintained during hospitalization for COVID-19 was related to a lower probability of ARDS (OR 0.78, 95% CI: 0.69–0.89 *p* < 0.001) and IMV (5.35 vs. 8.57%; *p* < 0.001) among 2,921 patients hospitalized for COVID-19 (58). In the study conducted by Gupta et al. (59) the statins use was associated with lower inpatient mortality (OR 0.47, 95% CI: 0.36–0.62, *p* < 0.001), but there were no differences in IMV or length of stay between the groups(statin users vs non-users). In another relevant cohort, The American Heart Association's COVID-19 Cardiovascular Disease Registry, Daniels et al. (60), evaluated the associations between statin use and different outcomes in 4,449 patients that were statin users prior to admission, alone or with anti-hypertensive drugs. Statins use was associated with a reduced risk of death (aOR 0.59, 95% CI 0.50–0.69). Moreover, it's important to highlight that a report from the same author showed that statin use during the 30 days before to admission for COVID-19 was associated with a decreased risk of developing severe COVID-19 and a faster time to recovery (61).

In a meta-analysis including four studies with a total of 8,990 COVID-19 patients from China, Italy or the USA, statin use significantly decreased the risk for fatal or severe disease (pooled HR 0.70; 95% CI: 0.53–0.94) (62). Similarly, another meta-analysis (seven observational studies; *n* = 2, 398 hospitalized COVID-19 patients from European and North American countries; *n* = 1,075 statin users) reported a significant association between a lower risk of progression to severe/critical illness or death in those patients taking statins (OR: 0.59; 95% CI: 0.35–0.99) (33). This statin-induced benefit was magnified after excluding studies in which statin treatment was initiated during hospitalization (OR 0.51; 95%CI: 0.41–0.64) (33). Of note, a favorable effect of statins on SARS-CoV-2 mortality was observed in a meta-analysis of 10 studies (aOR 0.73; *p* = 0.0237) (34). Conflicting results were reported for patients with diabetes (34).

In a retrospective study among 255 COVID-19 patients, pre-admission statin use significantly lowered the odds of in-hospital mortality by 86% (OR 0.14, 95% CI: 0.03–0.61, *p* = 0.008) (63). Permana et al. (64) performed a meta-analysis evaluating 13 studies with a total of 52,122 patients; 8 studies reported in-hospital administration of statins and the remaining 5 studies pre-admission statin use. In-hospital use of statin was related to a reduced risk of death [relative ratio (RR) 0.54, 95% CI: 0.50–0.58, *p* < 0.00001), but not pre-admission use (RR 1.18, 95% CI: 0.79–1.77; *p* = 0.415). In another meta-analysis of 14 observational studies (*n* = 19,988 patients with COVID-19), statin administration (prior and in-hospital) decreased the risk of adverse outcomes (OR 0.51; 95% CI: 0.41–0.63; *p* < 0.0005) (65).

Overall, there are studies showing that statins may favorably affect the course of COVID-19, thus highlighting the clinical importance of statin use in the COVID-19 era. Table 1 summarizes the meta-analyses evaluating the effects of statin use on outcomes in patients with COVID-19 (62, 64–67).

**Table 1:** Summary of meta-analyses evaluating the effects of statin use on outcomes in patients with COVID-19.

**Authors (Ref)**	**Study design**	**Population Health status**	**Sample size**	**Main findings Statin use vs. non-use**
Kow et al. (62)	Meta-analysis	COVID-19 patients in China, USA, and Italy	8,990	Fatal or severe disease COVID-19 (pooled HR 0.7, 95% CI: 0.53–0.94; *p* = 0.01)
Onorato et al. (66)	Meta-analysis	COVID-19 patients in Europe and North America	2,398	Progression to severe illness or death (OR 0.59, 95% CI: 0.35–0.99)
Scheen (67)	Meta-analysis	COVID-19 patients in China, USA, Europe, Japan, and Iran	42,722	Hard clinical outcomes (adjusted OR 0.73; *p* = 0.0237)
Permana et al. (64)	Meta-analysis	COVID-19 patients from in France, Spain, and USA	52,122	All-cause mortality (RR 0.54, 95% CI: 0.50–0.58; *p* < 0.00001)
Pal et al. (65)	Meta-analysis	COVID-19 patients in China, USA, Europe, Japan and Iran	19,988	Adverse COVID-19 outcomes (OR 0.51, 95% CI 0.41–0.63; *p* < 0.0005)

*COVID-19, Coronavirus disease; IMV, invasive mechanical ventilation; ARDS, acute respiratory distress syndrome; HR, hazard-ratio; OR, odds ratio; RR, relative risk; CI, confidence intervals*.

### Future Perspectives

Available human studies have shown beneficial effects of statins on pneumonia (7, 68) and sepsis. Indeed, in the Jupiter trial (*n* = 17,802) rosuvastatin showed a protective effect on incident pneumonia compared with placebo (HR: 0.83, 95% CI: 0.69-1.00) (39). There is experimental and animal data suggesting that statins can attenuate acute lung injury by decreasing pro-inflammatory cytokine release, endothelial dysfunction and thrombosis, effects that can improve pneumonia and sepsis outcomes (69). A meta-analysis found that statins may exert a neutral effect on sepsis (70). Therefore, and based on the paucity of robust randomized data, further research is needed to assess the impact of statin treatment on the incidence and outcomes of bacterial and viral infections, including the SARS-CoV-2.

Another issue is whether statin type and dose may differentially affect COVID-19 outcomes. This hypothesis was evaluated in a cohort of 71 patients with previous CV disease reporting that all-cause mortality was significantly reduced by lipophilic statins (atorvastatin in 22 patients and simvastatin in 4) but not with hydrophilic statins (rosuvastatin in 14 patients and pravastatin in 2) (71). Therefore, clinical trials are needed to elucidate the impact of different statins on the course of COVID-19.

Observational studies showed that statins may lower the rate of IMV and ARDS in COVID-19 patients. Furthermore, several meta-analyses found that statins can decrease mortality during SARS-CoV-2 infection in hospitalized patients. However, randomized controlled trials are required to verify these results in COVID-19 patients. In this context, several clinical trials are currently ongoing to evaluate the beneficial effects of statins in patients with COVID-19. These clinical trials are assessing the effect of statins (atorvastatin, rosuvastatin, simvastatin) alone (NCT04486508, NCT04359095, NCT04333407, NCT02735707, NCT04801940, NCT04380402, https://clinicaltrials.gov) or in combination with other drugs such as colchicine, nevibolol, folic acid, ruxolitinib or lopinavir/ritonavir (NCT04472611, NCT04631536, NCT04348695, NCT04466241, https://clinicaltrials.gov). The results of these studies will elucidate the role of statins in COVID-19 outcomes.

However, there are studies that show that the use of statins does not improve the prognosis of patients infected with SARS-CoV-2, specifically, Hariyanto et al. (72), performed a systematic review with 9 observational trials to evaluate the association between statin use and COVID-19 outcomes. Although statins users didn't improved outcomes, they emphasize that it is necessary to maintain this medication due to their cardiovascular benefits.

In conclusion, statins can exert several beneficial effects in terms of inflammation, endothelial dysfunction, lipid rafts, coagulation, thrombosis, ACE2 and CD147 expression and function. Through these mechanisms of action, statins may favorably affect the incidence and course of COVID-19. There are observational clinical data and meta-analyses supporting a protective impact of statin use in COVID-19 patients. Ongoing prospective randomized clinical trials will further elaborate the role of statins in SARS-CoV-2 infection.

## Author Contributions

JT-P and PP-M: conceptualization and writing—original draft preparation. JT-P, PP-M, and NK: literature review and writing—review and editing. PP-M and NK: supervision. All authors have read and agreed to the published version of the manuscript.

## Conflict of Interest

The authors declare that the research was conducted in the absence of any commercial or financial relationships that could be construed as a potential conflict of interest.

## Publisher's Note

All claims expressed in this article are solely those of the authors and do not necessarily represent those of their affiliated organizations, or those of the publisher, the editors and the reviewers. Any product that may be evaluated in this article, or claim that may be made by its manufacturer, is not guaranteed or endorsed by the publisher.
